# Perceptions of the population regarding generic drugs in Brazil: a nationwide survey

**DOI:** 10.1186/s12889-015-1475-1

**Published:** 2015-02-10

**Authors:** Elene P Nardi, Marcos B Ferraz, Geraldo RC Pinheiro, Sérgio C Kowalski, Emilia I Sato

**Affiliations:** São Paulo Center for Health Economics (GRIDES), Universidade Federal de São Paulo, Rua Botucatu 740, São Paulo, CEP- 04023-062 Brasil; Department of Internal Medicine, Discipline of Rheumatology, Universidade do Estado do Rio de Janeiro, Av 28 de Setembro, 77 - sala 333, Vila Isabel, CEP 20551-030 Rio de Janeiro, Brasil; Department of Clinical Epidemiology and Biostatistics, Mc Master University, 1280 Main St West, Office HSC 2C20, L8S 4K1 Hamilton, Ontario Canada; Department of Medicine, Rheumatology Division, Universidade Federal de São Paulo, Rua Botucatu 740, CEP- 04023-062 São Paulo, Brasil

**Keywords:** Drugs, Generic, Drugs, Nonproprietary, Perception, Brazil

## Abstract

**Background:**

Generic drugs (GDs) offer a way to reduce health spending without sacrificing quality. Despite this, there are doubts as to their acceptance by the population. This work aims to assess perceptions of GDs among the Brazilian population.

**Methods:**

We conducted a national household survey face-to-face between April and May 2013, with 5000 individuals aged over 15 years. The questions explored socioeconomic and demographic characteristics, the use of GDs, and perceptions about GDs as compared to brand drugs (BDs). The chi-square test was used to examine the associations between the perceptions and the characteristics of the population.

**Results:**

Of the 5000 participants, 51.3% were women, 40.2% were white, 48.6% were between 15 and 34 years of age, and 52.3% had income of less than two minimum wages (US$627.78). In terms of the use of GDs, 44.6% of the participants were taking or had taken GDs in the past three months, with the highest figures among the elderly (61.1%) and female (49.2%) populations. Regarding perceptions, 30.4% of the respondents considered GDs less effective than BDs; provided the same price, 59% would prefer BD, and 45.8% agreed that physicians prefer to prescribe GDs. The most negative perceptions about GDs were observed among lower income, elderly and nonwhite populations.

**Conclusion:**

The findings provide a better understanding of Brazilians’ perceptions regarding GDs. This should be considered when formulating healthcare policies aiming at improving access to effective and quality drugs, and reduction of health costs.

## Background

According to a survey of family budgets conducted in 2008-2009 by the Brazilian Institute for Geography and Statistics (IBGE), medicines account for around 48.6% of the total amount spent on healthcare by Brazilian families. Among low-income families, this proportion is as high as 74.2% [1]. In this context, generic drugs (GDs) offer a good alternative for reducing out-of-pocket spending by families, without sacrificing product safety or quality.

GDs were approved by the National Drug Policy, in October 1998, as a means of promoting rational use of medicines [2]. The measure was legally consolidated some months later, in February, 1999 [3]. While ensuring safety and quality, the GD policy aimed to provide drugs at lower prices [4]. Since then, the Market share of GDs in Brazil has grown steadily each year, and by November 2013, 3,591 generic drugs were registered with the Brazilian National Health Surveillance Agency (ANVISA) with 436 different active substances [4]. Nevertheless, generic drugs still have a small market share in Brazil (27.21% of total volume), particularly when compared with countries like Germany and the United Kingdom, which have market shares in 2011 of 76% and 75%, respectively [5,6].

Despite the lower price and the required proof of bioequivalence, the replacement and acceptance of generic drugs still raises questions. In 2001, a study by ANVISA showed that although 80% of respondents believed that GDs had the same effect as non-generic drugs, only 48% asked to replace brand drugs (BDs) for GDs [7]. A more recent study conducted in southern Brazil found that 33.8% of the 374 respondents either did not know about GDs, or considered them inferior products [8]. Even the United States, which has had regulation of GDs since 1984 (HatchWaxman Act - US Public Law 98-417), shows concerns about the acceptance of GDs and their perceptions in the market [9]. Two works published by William Shank reported that despite a positive attitude to generics, only about a third of the 1,047 interviewees preferred GDs, and almost half of the 506 physicians surveyed showed concerns about quality [10,11]. In addition, the existence of different types of drugs could cause confusion among general population and influence the perception regarding GDs, especially when it is considered that there are some differences in appearance (e.g. shape, color and size) between a BD and its GD.

Given the importance of generic drugs on the Brazilian market with limited financial resources for healthcare, this work aims to evaluate perceptions of the value and potential limitations with generic medication among the Brazilian population.

## Methods

This study was part of Brazilian Copcord Study (BRAZCO), a cross-sectional population based study conducted between April and May 2013. We surveyed 5,000 participants over the age of 15 from sixteen capitals of five regions of Brazil: North (Belém, Manaus), Northeast (Fortaleza, João Pessoa, Maceió, Natal, Recife and Salvador), Southeast (Belo Horizonte, Rio de Janeiro and São Paulo), South (Curitiba, Florianópolis and Porto Alegre), and Midwest (Brasília and Goiânia).

The sample was comprised of representative quotas of the Brazilian population, proportional to the population densities of the capitals in each region of the country, based on the Census conducted in 2010 by the IBGE. The quotas of gender and age in each capital were based on the Census, and participants of all socioeconomic statuses, educational levels and occupations were included.

The selection of households was random, with systematic selection of streets and subjects by randomly drawing the census tract with quota control for the seasonality factor. Taking the list of households, one household was evaluated per street, up to a total of ten households in the sector. If an entire sector was covered but not enough households were found to complete the required number, the process was re-started in the sector, beginning in the first street, five houses after the house of the first interview. In each household, up to three visits were made, on different days and times. In cases where the interview couldn’t be made after these three attempts, the household was replaced by another in the same Census sector. If the resident of the selected household could not be interviewed, that household was replaced by another in the same Census Sector, seeking to ensure a respondent with the same gender and age group.

A success rate of 70% was established, so 42.9% more households than planned were randomly selected to ensure substitution. Ineligible households, such as collective households (vacant households, hotels, lodges, nursing homes, etc.), agricultural, educational and healthcare establishments, and buildings under construction, were replaced by another in the same Census sector. The maximum sampling error was ±1.39% for the country as a whole, with a 95% confidence level.

Residents who did not speak Portuguese, and people with cognitive disability being incapable of reliably and consistently answering the questionnaire were excluded. Because the minor proportion of people living in rural areas (15.6%) and the difficulty of access this scattered population, only households in urban area were considered [12].

The survey instrument was a household questionnaire conducted face-to-face by a specialized team, consisting of open and closed-ended questions about socioeconomic and demographics aspects, and the use of GDs in the past three months. Race was declared by the respondents themselves (black, mixed race, Asian, or Indigenous) and then classified as white or nonwhite. Family income was expressed as multiples of minimum wages, where the values of the Brazilian minimum wage, originally in Reais (Brazilian currency), were converted to United States dollars (US$) according to the exchange rate of 2013 using data from Institute of Applied Economic Research (IPEA – Instituto de Pesquisa Econômica Aplicada) [13].

To evaluate the perceptions on GDs as compared to BDs, four multiple choice questions (agree, disagree or don’t know) were presented to the participants, concerning effectiveness, use, individual preference and physicians’ preferences. To reduce bias among the respondents regarding their opinions about GDs, two sentences concerning the BDs were included: “Brand drugs cause more side effects (adverse reactions) than generic drugs” and “Brand drugs take more time to the effect when compared to generic drugs”. The statements tried to avoid systematically favor or disfavor BDs or GDs.

The questionnaires were reviewed by an independent supervisor and submitted to a process of evaluation of consistency, where 50% of the questionnaires were double checked through phone calls.

Descriptive statistics were used to examine the characteristics of the participants. The Chi-square test was used to examine the associations between the perception and the characteristics of the population. Statistical analyses were performed using the Minitab software, version 16, and statistical significance was assumed for p-values less than or equal to 0.05.

All participants were informed about the study and signed a consent form. The study protocol was reviewed and approved by the Research Ethics Committee of the Federal University of São Paulo.

## Results

Table 1 presents the main demographics and socioeconomic characteristics of the surveyed population, and the number of participants who were taking or had taken GDs in the past three months.

**Table 1:** **Demographics and socioeconomic characteristics of the Brazilian population, study sample (n = 5000) and use of generic drugs**

	**Brazilian population** ^**a**^		**Participants**	
	**N**	**%**	**N**	**%**
**Total**	190 755 799	-	5000	**-**
**Gender**				
Male	93 406 990	49.0	2433	48.7
Female	97 348 809	51.0	2567	51.3
**Age (years)**				
15 to 34	67 084 990	46.3	2430	48.6
35 to 64	63 657 038	44.0	2097	41.9
≥65	14 081 477	9.7	473	9.5
**Region**				
North	15 864 454	8.3	415	8.3
Northeast	53 081 950	27.8	1390	27.8
Midwest	14 058 094	7.4	370	7.4
Southeast	80 364 410	42.1	2105	42.1
South	27 386 891	14.4	720	14.4
**Race**				
White	91 051 646	47.7	2009	40.2
Nonwhite	99 697 545	52.3	2991	59.8
**Marital status** ^**b**^				
Single	89 653 403	55.3	2053	41.1
Married	56 435 253	34.8	1632	32.6
Living common law	_		810	16.2
Widowed	8 063 404	5.0	240	4.8
Divorced/Separated	7 829 238	4.8	233	4.7
Did not answer	-	-	32	0.6
**Family income (minimum wages)** ^**b**^				
Less than 2 (US$ 627.78)	75 073 409	46.3	2615	52.3
From 2 – 10	24 140 811	14.9	2079	41.6
More than 10 (US$3,138.89)	2 686 709	1.7	218	4.4
Did not answer	-	-	88	1.7
**Was taking or had taken GD in the past three months**				
Yes	-	-	2230	44.6

**Note:** ^**a**^Source: Instituto Brasileiro de Geografia e Estatística – IBGE, Censo 2010: http://www.ibge.gov.br/home/estatistica/populacao/censo2010/default_resultados_universo.shtm.

**Note:** ^**b**^ ≥ 10 years (n = 161 981 299).

A total of 5000 participants from 16 capitals of five Brazilian geographic regions were surveyed: 415 participants from the North (8.3%), 1390 from the Northeast (27.8%), 2105 from Southeast (42.1%), 720 from South (14.4%), and 370 from Midwest (7.4%). Of the participants, n = 2567 (51.3%) were women, and 40.2% (n = 2009) described themselves as white. The participants were predominantly between 15 and 34 years old (48.6%) and 52.3% had a family income of up to two minimum wages, or US$627.78.

Of all the participants, 44.6% were taking or had taken GDs in the past three months. Table 2 describes the use of generic drugs, according to the characteristics of population. The highest use of GDs in the past three months was among females (49.2%, p < 0.001) and in the population over the age of 65 (61.1%, p < 0.001).

**Table 2:** **Proportion of peoples who were taking or had taken GD in the past three months**

	**Was taking or had taken GD in the past three months**	
	**N (%)**	**p-value**
**All**	2230 (44.6)	
**Gender**		
Male	968 (39.8)	<0.001
Female	1262 (49.2)	
**Age**		
15 - 34 years	935 (38.5)	<0.001
35 - 64 years	1006 (48.0)	
≥65 years	289 (61.1)	
**Race**		
White	906 (45.1)	0.562
Nonwhite	1324 (44.3)	
**Family income (minimum wages)** ^**a**^		
Less than 2 (US$ 627.78)	1159 (44.3)	0.380
2 a 10	938 (45.1)	
More than 10 (US$3,138.89)	107 (49.1)	
Did not answer	26 (29.5)	

***Note:*** ^***a***^ *Only included those who reported family income during the interview.*

Tables 3 and 4 show the perceived value attributes expressed by all the participants, of GDs when compared to BDs, stratified by some demographics and economic characteristics of the sample.

**Table 3:** **Perception of value attributes of generic drugs according to gender, age and use of generic drugs**

		**All (n = 5000)**	**Gender**		**Age (years)**			**Was taking or had taken GD in the past three months**	
	**N(%)**		**Male**	**Female**	**15-34**	**35-54**	**>54**	**Yes**	**No**
**Generic drugs are less effective (less powerful) than brand name drugs**	**I agree**	1520 (30.4)	741 (30.5)	779 (30.3)	660 (27.2)	521 (32.4)	339 (35.2)	642 (28,8)	878 (31,7)
	**I disagree**	2942 (58.8)	1393 (57.3)	1549 (60.3)	1526 (62.8)	918 (57.1)	498 (51.7)	1404 (63,0)	1538 (55,5)
	**I don’t know/Did not answer**	538 (10.8)	299 (12.3)	239 (9.3)	244 (10.0)	168 (10.5)	126 (13.1)	184 (8,3)	354 (12,8)
	**p-value** ^**a**^		0.375		<0.001			<0.001	
**Brand drugs cause more side effects (adverse reactions) than generic drugs.**	**I agree**	1403 (28.1)	694 (28.5)	709 (27.6)	670 (27.6)	463 (28.8)	270 (28.0)	619 (27,8)	784 (28,3)
	**I disagree**	2798 (56.0)	1328 (54.6)	1470 (57.3)	1396 (57.4)	914 (56.9)	488 (50.7)	1283 (57,5)	1515 (54,7)
	**I don’t know/Did not answer**	799 (16.0)	411 (16.9)	388 (15.1)	364 (15.0)	230 (14.3)	205 (21.3)	328 (14,7)	471 (17,0)
	**p-value** ^**a**^		0.220		0.275			0.287	
**Brand drugs take more time to the effect when compared to generic drugs**	**I agree**	632 (12.6)	325 (13.4)	307 (12.0)	308 (12.7)	192 (12.0)	132 (13.7)	261 (11,7)	371 (13,4)
	**I disagree**	3672 (73.4)	1748 (71.8)	1924 (74.9)	1789 (73.6)	1233 (76.7)	650 (67.5)	1698 (76,1)	1974 (71,3)
	**I don’t know/Did not answer**	696 (13.9)	360 (14.8)	336 (13.1)	333 (13.7)	182 (11.3)	181 (18.8)	271 (12,2)	425 (15,3)
	**p-value** ^**a**^		0.076		0.097			0.021	
**Generic drugs are more suitable or appropriate than brand name drugs for mild, banal or less serious diseases.**	**I agree**	2049 (41.0)	1024 (42.1)	1025 (39.9)	974 (40.1)	670 (41.7)	405 (42.1)	904 (40,5)	1145 (41,3)
	**I disagree**	2344 (46.9)	1106 (45.5)	1238 (48.2)	1181 (48.6)	772 (48.0)	391 (40.6)	1104 (49,5)	1240 (44,8)
	**I don’t know/Did not answer**	607 (12.1)	303 (12.5)	304 (11.8)	275 (11.3)	165 (10.3)	167 (17.3)	222 (10,0)	385 (13,9)
	**p-value** ^**a**^		0.065		0.023			0.048	
**Physicians prefer to prescribe generic drugs.**	**I agree**	2289 (45.8)	1032 (42.4)	1257 (49.0)	1160 (47.7)	706 (43.9)	423 (43.9)	1051 (47,1)	1238 (44,7)
	**I disagree**	2090 (41.8)	1035 (42.6)	1055 (41.1)	978 (40.3)	708 (44.1)	404 (42.0)	971 (43,5)	1119 (40,4)
	**I don’t know/Did not answer**	621 (12.4)	366 (15.0)	255 (9.9)	292 (12.0)	193 (12.0)	136 (14.1)	208 (9,3)	413 (14,9)
	**p-value** ^**a**^		0.003		0.032			0.718	
**If there was not a price difference (generics are cheaper), I would always prefer taking a brand name drug.**	**I agree**	2962 (59.2)	1438 (59.1)	1524 (59.4)	1403 (57.7)	994 (61.8)	565 (58.7)	1363 (61,1)	1599 (57,7)
	**I disagree**	1666 (33.3)	780 (32.1)	886 (34.5)	847 (34.9)	504 (31.4)	315 (32.7)	743 (33,3)	923 (33,3)
	**I don’t know/Did not answer**	372 (7.4)	215 (8.8)	157 (6.1)	180 (7.4)	109 (6.8)	83 (8.6)	124 (5,6)	248 (9,0)
	**p-value** ^**a**^		0.258		0.044			0.352	

***Note*** *:* ^*a*^ *Only included those who answered the statements.*

**Table 4:** **Perception of value attributes of generic drugs according to race and family income**

		**All (n = 5000)**	**Race**		**Family Income (minimum wages)** ^**a**^			
	**N(%)**		**White**	**Nonwhite**	**1 - 2**	**2 - 10**	**>10**	**Did not answered**
**Generic drugs are less effective (less powerful) than brand name drugs**	**I agree**	1520 (30.4)	545 (27.1)	975 (32.6)	848 (32.4)	598 (28.8)	53 (24.3)	21 (23.9)
	**I disagree**	2942 (58.8)	1258 (62.6)	1684 (56.3)	1456 (55.7)	1291 (62.1)	140 (64.2)	55 (62.5)
	**I don’t know/Did not answer**	538 (10.8)	206 (10.3)	332 (11.1)	311 (11.9)	190 (9.1)	25 (11.5)	12 (13.6)
	**p-value** ^**b**^		<0.001		<0.001			
**Brand drugs cause more side effects (adverse reactions) than generic drugs.**	**I agree**	1403 (28.1)	444 (22.1)	959 (32.1)	861 (32.9)	492 (23.7)	33 (15.1)	17 (19.3)
	**I disagree**	2798 (56.0)	1243 (61.9)	1555 (52.0)	1323 (50.6)	1272 (61.2)	146 (67.0)	57 (64.8)
	**I don’t know/Did not answer**	799 (16.0)	322 (16.0)	477 (15.9)	431 (16.5)	315 (15.2)	39 (17.9)	14 (15.9)
	**p-value** ^**b**^		<0.001		<0.001			
**Brand drugs take more time to the effect when compared to generic drugs**	**I agree**	632 (12.6)	209 (10.4)	423(14.1)	382 (14.6)	221 (10.6)	20 (9.2)	9 (10.2)
	**I disagree**	3672 (73.4)	1514 (75.4)	2158 (72.1)	1856 (71.0)	1589 (76.4)	173 (79.3)	54 (61.4)
	**I don’t know/Did not answer**	696 (13.9)	286 (14.2)	410 (13.7)	377 (14.4)	269 (13.0)	25 (11.5)	25 (28.4)
	**p-value** ^**b**^		<0.001		<0.001			
**Generic drugs are more suitable or appropriate than brand name drugs for mild, banal or less serious diseases.**	**I agree**	2049 (41.0)	683 (34.0)	1366 (45.7)	1164 (44.5)	789 (38.0)	80 (36.7)	16 (18.2)
	**I disagree**	2344 (46.9)	1074 (53.5)	1270 (42.5)	1120 (42.8)	1054 (50.7)	112 (51.4)	58 (65.9)
	**I don’t know/Did not answer**	607 (12.1)	252 (12.5)	355 (11.9)	331 (12.7)	236 (11.3)	26 (11.9)	14 (15.9)
	**p-value** ^**b**^		<0.001		<0.001			
**Physicians prefer to prescribe generic drugs.**	**I agree**	2289 (45.8)	851 (42.4)	1438 (48.1)	1317 (50.4)	896 (43.1)	62 (28.4)	14 (15.9)
	**I disagree**	2090 (41.8)	886 (44.1)	1204 (40.3)	981 (37.5)	959 (46.1)	120 (55.1)	30 (34.1)
	**I don’t know/Did not answer**	621 (12.4)	272 (13.5)	349 (11.7)	317 (12.1)	224 (10.8)	36 (16.5)	44 (50.0)
	**p-value** ^**b**^		<0.001		<0.001			
**If there was not a price difference (generics are cheaper), I would always prefer taking a brand name drug.**	**I agree**	2962 (59.2)	1114 (55.5)	1848 (61.8)	1609 (61.5)	1201 (57.8)	127 (58.3)	25 (28.4)
	**I disagree**	1666 (33.3)	723 (36.0)	943 (31.5)	827 (31.6)	731 (35.2)	73 (33.5)	35 (39.8)
	**I don’t know/Did not answer**	372 (7.4)	172 (8.6)	200 (6.7)	179 (6.9)	147 (7.1)	18 (8.3)	28 (31.8)
	**p-value** ^**b**^		<0.001		0.028			

***Note*** *:* ^*a*^ *Only included those who reported family income during the interview;* ^*b*^ *Only included those who answered the statements.*

The majority of participants (58.8%) disagreed with the statement that “GDs are less effective than BDs”. Older and nonwhite participants expressed less disagreement than younger participants (p < 0.001) and white (p < 0.001) participants, respectively. Participants who was taking or had taken GD in the past three months also disagreed more than those who had not (p < 0.001). Lower family income was associated with less disagreement with this statement than higher income (p < 0.001). However, approximately one third of all participants (30.3%) agreed that GDs are less effective than BDs. The level of agreement ranged from 24.3% in the high income group to 35.2% in the group of people aged over 54.

The majority of participants (56.0%) disagreed with the statement that “BDs cause more side effects (adverse effects) than GDs”. Nonwhite participants also expressed less disagreement with this statement (p < 0.001) than white participants. Again, higher income was correlated with more disagreement (p < 0.001). On the other hand, 28.1% of the entire sample agreed with this statement ranging from an agreement by 15.1% of the highest income group to an agreement of 32.6% of the nonwhite population.

Regarding the statement “BDs take more time to the effect than GDs”, 73.4% of the participants did not agree with it. This trend was observed in all subgroups, however a statistically significant increase in the level of disagreement was observed among higher family income groups (p < 0,001). The overall agreement with this statement was 12.6%.

When asked about their perceptions of the statement “GDs are more suitable or appropriate than BDs for mild or less serious diseases”, a slightly higher percentage of participants (46.9%) disagreed with this statement. This disagreement was higher among white participants (53.5%, p < 0.001) and in the higher family income groups (p < 0.011). The overall agreement with this statement was 41% with the highest level of agreement in the nonwhite population (45.7%) and the lowest level of agreement in the white population (34%).

A slightly higher proportion of participants (45.8%) agreed with the statement “Physicians prefer to prescribe GDs”. Females (49.0%) and the lowest family income group (50.4%) tended to agree more with it (p < 0.001). A disagreement was indicated by 41.8% of the total sample with the highest level of disagreement in the highest income group (55.1%) and the lowest disagreement among the lowest income participants (37.5%).

Finally, the majority of participants (59.2%) agreed with the statement “If there were no price difference (GDs are cheaper), I would always prefer to take a BD”. Comparing all the subgroups tested, only the nonwhite group expressed a greater and relevant disagreement (31.5%) with this statement when compared to the white group (36%; p < 0.001). Other observed statistically significant differences between subgroups were very small and therefore not important.

## Discussion

This work was the first nationwide survey evaluating the use and perception of GDs among the Brazilian population. Given that cost and reliance on drugs have been suggested in previous studies as key factors influencing adherence to medication, it is important to reduce individual healthcare costs in order to increase patients’ adherence to drug therapy [14].

Rural area residents were excluded from the sample, because of its minor proportion comparing to total of Brazilian population (15.6%) and the difficulty of access this scattered population [12]. It is worth emphasizing that, on the last Census, the quotas of gender and age in rural areas are similar to the urban population: (i) Urban – Male 48.3% and Female 51.7%; Rural – Male: 52.6% and Female: 47.4%; (ii) Urban - 15 to 34 years: 46.2%, 35 to 64 years: 44.2%, ≥65 years: 9.6%, and Rural - 15 to 34 years: 46.8%, 35 to 64 years: 42.8%, ≥65 years: 10.5% [12].

In this study, GDs have or had been used by 44.6% of the population within the past three months. This figure is higher than the frequency found in 2006/2007 in an area covered by a family health unit in a city in southern Brazil (9.9%). It is also higher than that found in another southern Brazilian city in 2002 (3.6%) [8,15]. These differences can be explained by the three month period used in the present study, as compared with the 7 and 15 days, respectively, used in the previous studies. Moreover, it can be explained by the ease of access to these drugs, since the market share of GDs has increased almost three-fold in Brazilian healthcare centers throughout the past five years [5].

Higher use of GDs was observed in the female and elderly populations, which can be explained by the higher use of medicines in general, by these two groups [16,17]. The higher use of GDs among the elderly population may also be related to their lower spending power, which may cause a preference for cheaper products like GDs. In August 2013, GDs were, on average, 56.63% cheaper than their respective brand name drugs (BDs) in Brazil [18].

Regarding the participants’ perceptions of the attributes of GDs, it was observed that almost a third (30.4%) believed GDs were less effective than BDs, and the most negative opinions were observed in the lower income, elderly and nonwhite populations. These results are consistent with previous studies, which found unfavorable opinions on the effectiveness, safety, tolerability and acceptance of GDs associated with low income, nonwhite race and advanced age of the respondents [11,19-22]. This raises a concern as to the acceptance of GDs and the adherence to drug therapy in these groups.

Additionally, participants who was taking or had taken GD in the past three months disagreed more with the statement that GDs were less effective than BDs. It is possible to assume that experience with GDs could possibly influence attitudes in a positive way.

In 2001 the National Health Surveillance Agency (ANVISA) surveyed 2,220 customers in 236 different cities, and observed that 80% of the participants were confident that GDs had the same effect as BDs [23]. A study conducted in the state of Rio Grande do Sul in 2002 found that 70% of the 3,182 participants believed GDs had similar quality compared to BDs, and a study conducted in Paraná in 2011 showed that 64.3% of the 374 participants were of the same opinion [8,15]. In this study, the majority of 58.8% of the 5,000 participants disagreed that GDs are less effective than BDs. This may represent a decline in the credibility of GD over the years. On the other hand, there were several differences between the surveys which may explain the variation: this study covered a greater population and more regions than previous studies and used a different survey approach concerning questions and methodology.

Although most of participants had a positive perception regarding the effectiveness of GDs, 59% said that if there were no price difference, they would prefer BDs. This finding reinforces the idea that price has a strong influence on the decision to purchase a GD, which was observed in a study conducted in the North of Brazil that evaluated the social representations of GDs by consumers and demonstrated price as a fundamental element in building product value and guiding market choice [24].

The findings indicate an underuse of GDs, with 41% of the participants agreeing with the statement *“Generic drugs are more suitable or appropriate than brand name drugs for mild, banal or less serious diseases”.* This result is in line with previous findings that participants would be more willing to use generics for less serious diseases, and it suggests that they may be more reluctant to use them for more serious diseases [21,25,26].

Another important finding is that a significant portion of the population agreed that BDs cause more side effects than GDs (28.1%). This proportion increased to almost a third when we evaluated only nonwhite and low-income populations. One possible explanation for this result is that some participants may see generics as products with reduced effectiveness, compared with brand name drugs, because they consider GDs to be less effective. Thus, BDs are perceived as stronger medications, and would cause more side effects than generics. The work, conducted by Sewell (2012), also indicated a perception regarding GDs as a weaker product. However, Sewell observed that GDs were perceived as having to be stronger to produce the same effect as BDs, leading to more side effects, contrary to what we found in the present study [26]. However, the expectation for more adverse events with BDs may be due to the perceived higher strength. More studies, and a better understanding regarding the population’s perceptions, are needed to confirm this finding and the underlying reasons.

Given the importance of physicians’ views as opinion leaders in the use of GDs, we also evaluated the population’s perceptions regarding the preference of these professionals. Less than half of the participants agreed with the statement that physicians prefer to prescribe generic drugs (45.8%). Moreover, it was observed that the low-income participants were more of the opinion that physicians prefer to prescribe GDs than the higher income population. We consider this difference by income to be positive, since this result indicates that physicians take socioeconomic criteria into account when making prescriptions. Another explanation is the greater use of the public health system by people with low incomes. According the National Sample Survey of Households (PNAD) conducted in 2008 by the IBGE, and a study by Zilda Pereira da Silva (2006), the population belonging to the first quintile (poorest 20%) is the main user of the public health system in Brazil [27,28]. Physicians from the public health system are obliged to prescribe drugs by their Brazilian Common Denomination (*Denominação Comum Brasileira* - DCB) or, in its absence, by the International Nonproprietary Name (INN) [29]. In these cases, users of public health system received more prescriptions for GDs than users of private health systems.

Evaluating perceptions on the attributes of generic drugs by gender, we found no statistically significant differences between the responses of women and men, except that women agreed more with the statement that physicians prefer to prescribe GDs. This finding differs from the study conducted by Shank (2009), which observed that women were more likely than men to report that generics offer better value than brand name drugs [11]. A potential explanation for the perceived higher prescribing of GDs among the women could be that a higher percentage of women tends to be in the lower income class and thus, more women may depend on the public system [30]. This effect would be even more pronounced for the differences between races [31].

The present work investigated the population’s perception regarding GDs, but did not investigate the reasons for the perceptions. It is necessary to explore the underlying reasons, for instance, if the negative perception that part of population has regarding GD is caused by lack of knowledge between the two types of drugs. Moreover, it is necessary to investigate whether the regulation and health surveillance concerning GDs in Brazil are appropriate to ensure effectiveness and interchangeable products with assured quality.

It is also necessary to educate the general public with regard to correct and conscious use of the different types of drugs. As suggested by a study conducted by Joan-Antoni Vallès (2002) in Spain, verbal information and the distribution of explanatory material about GDs for patients will increase their use [32]. However, further studies are needed to confirm the importance of patient education in the choice of drugs in Brazil.

## Conclusions

Our findings demonstrate that although the majority of participants have a positive attitude towards GDs, there is a considerable percentage who expressed concerns about these drugs, particularly the older, low income and nonwhite populations. That more negative attitudes towards GDs are observed with elderly and low income populations is in contrast to their greater need for affordable products. A perceived inferiority of generics and higher cost of branded drugs may lead to lower adherence to drug therapy among these population groups.

A better understanding of individual perceptions regarding GDs can stimulate actions that will improve the effective use of generic drugs by the Brazilian population and guide the formulation of appropriate health policies aimed at increasing access to effective, safe and affordable drugs.

### Ethical standards

The experiment complies with the current laws of the country in which it was performed.

## Abbreviations

ANVISA: Brazilian national health surveillance agency (Agência Nacional de Vigilância Sanitária)
BDs: Brand drugs
BRAZCO: Brazilian Copcord study
GDs: Generic drugs
IBGE: Brazilian institute for geography and statistics (Instituto Brasileiro de Geografia e Estatística)
